# C-Reactive Protein Is an Indicator of the Immunosuppressive Microenvironment Fostered by Myeloid Cells in Hepatocellular Carcinoma

**DOI:** 10.3389/fonc.2021.774823

**Published:** 2022-01-06

**Authors:** Yongchun Wang, Zhixiong Li, Zhijie Huang, Xingjuan Yu, Limin Zheng, Jing Xu

**Affiliations:** ^1^ Collaborative Innovation Center for Cancer Medicine, State Key Laboratory of Oncology in South China, Sun Yat-sen University Cancer Center, Guangzhou, China; ^2^ Ministry Of Education (MOE) Key Laboratory of Gene Function and Regulation, School of Life Science, Sun Yat-sen University, Guangzhou, China

**Keywords:** CRP, HCC, immune microenviroment, tumor associated macrophage, tumor associated neutrophil

## Abstract

**Background:**

C-reactive protein (CRP) is a widely used marker of systemic inflammation and predicts poor clinical outcomes in patients with hepatocellular carcinoma (HCC); however, its significance in the local immune response at the tumor site is not clear.

**Methods:**

Serum CRP levels of 329 HCC patients were detected before resection. Paired paraffin-embedded tumor samples were used to quantify immune cell populations, such as CD11b^+^ myeloid cells, CD68^+^ macrophages (Mφs), CD15^+^ neutrophils, CD8^+^ T cells, and CD206^+^, CD204^+^, CD163^+^ and CD169^+^ Mφs, by immunohistochemistry. Enrichment scores for 34 types of immune cells based on transcriptome data from 24 HCC samples were calculated by xCell. Overall survival of patients was analyzed using the Kaplan-Meier method.

**Results:**

Serum CRP levels were correlated with liver functions and tumor stages in patients with HCC. The densities of CD68^+^ tumor-associated macrophages (TAMs) and CD15^+^ tumor-associated neutrophils (TANs) were significantly higher in patients with elevated serum CRP levels than in those with low CRP levels (both *p <* 0.0001). Further analysis of TAM subtypes revealed that serum CRP levels were associated with CD204^+^ and CD163^+^ Mφ densities (*p* < 0.0001 and *p* = 0.0003, respectively). Moreover, transcriptome data showed that CRP expression was associated with the expression of myeloid cell infiltration-related genes in HCC tumors. The combination of serum CRP with TAMs or TANs in both the nontumor and intratumor regions could represent a powerful criterion for predicting patient prognoses.

**Conclusion:**

Serum CRP could serve as an indicator of an immunosuppressive TME in HCC, which could be of potential clinical application for treatment strategies targeting the TME.

## Introduction

Hepatocellular carcinoma (HCC) is one of the most common neoplasms worldwide and is associated with an increased incidence and extremely poor prognosis (1). HCC is usually found in inflamed fibrotic and/or cirrhotic livers with extensive immune infiltration due to chronic viral infection. The immune status at a tumor site can largely influence the biologic behavior of tumors. However, there is still a lack of easy-to-use clinical markers to reflect the local immune response of HCC.

C-reactive protein (CRP) is a highly sensitive marker of systemic inflammation that is mainly released by hepatocytes in response to tissue injury, infection or inflammation. The circulating concentration of CRP is typically < 2 mg/L in healthy individuals and often increases to a high level in the presence of inflammation (2). Elevated levels of serum CRP have been associated with an increased risk of several chronic conditions, such as cardiovascular disease, atherosclerosis, and cancer (3). In patients with HCC, increased levels of CRP are correlated with reduced survival (4, 5). While the association between CRP and systemic inflammation in cancer patients is generally accepted (3–7), little is known about the significance of serum CRP levels and the local immune status of the tumor.

Leukocyte infiltration constitutes a major component of the tumor stroma, forming a highly complex, dynamic, and interactive immune contexture, which contributes to tumor development and progression (8, 9). Many of these infiltrating cells, including T cells, myeloid-derived suppressor cells (MDSCs), tumor-associated macrophages (TAMs) and tumor-associated neutrophils (TANs), are educated by tumor environmental signals and often acquire special phenotypic characteristics during tumor progression (8–10). Our previous studies showed that myeloid cells secrete various cytokines, such as IL-1β and TNF-α, to promote tumor cell autophagy and angiogenesis and correlate with poor clinical outcome in HCC patients (11–14). It has been reported that tumor and stromal cells, including TAMs and TANs, secreting inflammatory IL-1 and IL-6 could be responsible for the elevated synthesis and secretion of CRP by hepatocytes (4, 5). This evidence prompted us to hypothesize that the level of serum CRP might correlate with the immune context at the tumor site of HCC patients.

To explore the relationship between CRP and the local immune response of HCC, we examined the association between serum CRP and various immune cell infiltrates in the nontumor and intratumor regions of HCC tissues. Our results showed that patients with elevated serum CRP levels tended to have more immunosuppressive TAM and TAN infiltrates, which are important contributors to the tumor-promoting milieu and facilitate tumor progression. These results suggested that the serum CRP level might be a potential indicator of an immunosuppressive microenvironment in HCC tumors.

## Patients and Methods

### Patients

A total of 329 HCC patients with complete follow-up data who underwent curative resection from 2010 to 2016 at Sun Yat-sen University Cancer Center were randomly enrolled in this study. None of the patients had received anticancer therapies before sampling. Individuals with a concurrent autoimmune disease, HIV, or syphilis were excluded. The preoperative blood examinations including serum CRP were done within 2 days prior surgery. Tumor samples from 24 patients who received therapy were used for isolation of RNA and microarray assay analysis. Clinical stages were classified according to the guidelines of the International Union against Cancer. Macroscopic vascular invasion was determined using imaging or histology. Overall survival (OS) was defined as the interval between the dates of surgery and death. All samples were coded anonymously in accordance with local ethical guidelines, as stipulated by the Declaration of Helsinki with written informed consent and a protocol approved by the Review Board of Sun Yat-sen University Cancer Center.

### Immunohistochemistry

Paraffin-embedded and formalin-fixed HCC samples were cut into 5-μm sections, which were then processed for immunohistochemistry as previously described. Briefly, the sections were incubated with Abs against the following human antigens: CD8 (rabbit monoclonal, clone EP334, ZSBio, China), CD11b (rabbit monoclonal, clone EPR1344, Abcam, UK), CD68 (mouse monoclonal; clone PG-M1; Dako Cytomation, USA), CD15 (mouse monoclonal; clone LeuM1; ZSBio), CD206 (mouse monoclonal, clone 685645, R&D Systems, USA), CD204 (mouse monoclonal, clone SRA-C6, Transgenic, Japan), CD163 (mouse monoclonal, clone 10D6, ZSBio) and CD169 (sheep polyclonal, clone NS0, R&D Systems). Then, the sections were stained in an EnVision System (Dako Cytomation, USA). Positive cells were detected by microscopy and quantified using the Vectra-Inform image analysis system (Perkin-Elmer Applied Biosystems, Foster City, CA, USA).

### Statistics

Statistical analyses were performed using SPSS 24.0 software (IBM, Chicago, IL, USA) and GraphPad Prism 7 software (GraphPad Software, Inc, San Diego, CA). The *ggplot2* package of R and the *Pheatmap* package of R were used for heatmap display. Gene expression data of 366 HCC patients were downloaded from TCGA. Categorical variables are presented as percentages and compared using the chi-square (*χ*2) test or Fisher’s exact test. Continuous variables are presented as the mean ± standard error of the mean (SEM) and compared using Student’s *t*-tests. The Kaplan-Meier method was used to estimate the survival rates for the different groups, and differences in survival were compared using the log-rank test. Cumulative survival time was estimated using the Kaplan-Meier method, and *p* < 0.05 was considered statistically significant for all tests.

## Results

### Correlation of Serum CRP Level and Patient Characteristics

**Figure 1:**
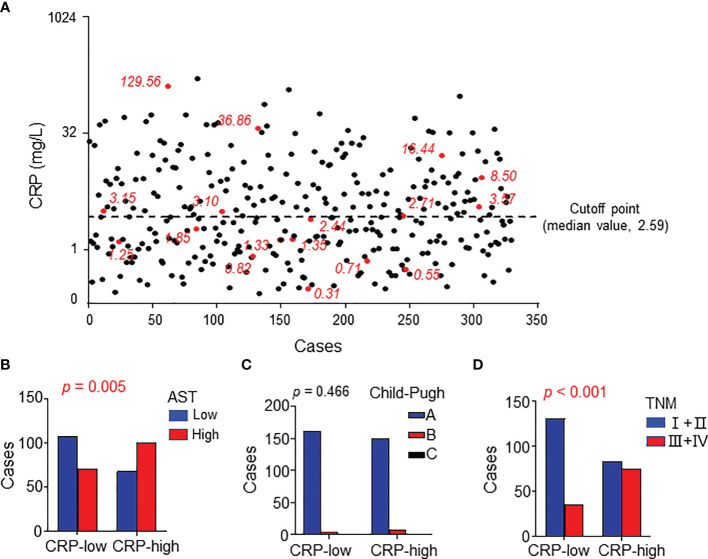
Serum CRP levels correlated with characteristics of HCC patients. **(A)** Serum CRP levels of 329 HCC patients. Red points represent the 17 samples examined in expression microarray analysis. The black dotted line indicates the median value as the cutoff for dividing patients into high or low groups. **(B–D)** The association between serum CRP and AST levels **(A)**, Child-Pugh **(B)**, and TNM stage **(C)**. Chi-square tests or Fisher’s exact tests were used, and *P* < 0.05 was considered statistically significant.

The patients’ clinical characteristics were summarized in **Table 1**. Considering that serum CRP is mainly synthesized by hepatocytes, we analyzed the correlation between serum CRP levels and liver function. The patients were divided into two groups according to the median CRP level (**Figure 1A**) and the results showed that CRP level significantly correlated with the circulating level of glutamic-oxaloacetic transaminase (AST, *p* = 0.005; **Figure 1B** and **Supplementary Table 1**). However, no significant difference was found between CRP levels and Child-Pugh score (**Figure 1C**), which might be due to the limited cases of Child-Pugh B (12/329) and C (1/329) in the study cohort. Moreover, the serum level of CRP was positively correlated with TNM stage (*p* < 0.001; **Figure 1D**), suggesting that an elevated level of serum CRP was associated with disease progression.

**Table 1:** Characteristics of patients.

Characteristics	No. (%)
Age, yr	
median	50
range	22-78
Gender	
male	288 (87.5)
female	41 (12.5)
HBV infection	
no	46 (14.0)
yes	239 (72.6)*
HCV infection	
no	312 (96.0)
yes	3 (0.9)*
Alpha-fetoprotein	
≤25 ng/ml	122 (37.1)
>25 ng/ml	196 (59.6)*
Child-Pugh score	
A	305 (92.7)
B	12 (3.6)
C	1 (0.3)*
Tumor number	
single	243 (75.0)
multiple	79 (24.4)*
Tumor size	
≤5 cm	93 (28.3)
>5 cm	231 (70.2)*
TNM stage	
I-II	213 (64.7)
III-IV	105 (31.9)*

*Information of the indicated characteristics is not available for some patients.

### Circulating CRP Level and Immune Infiltration in HCC

**Figure 2:**
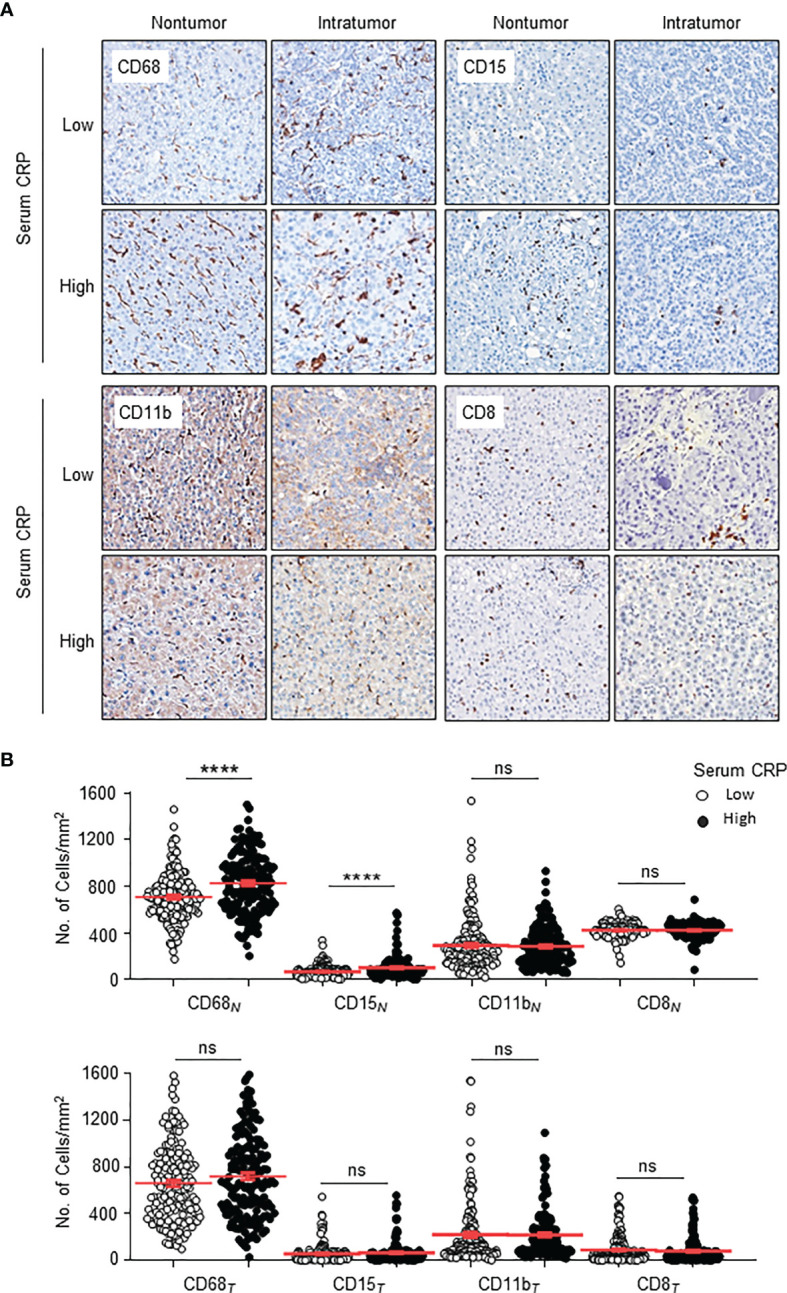
Distributions of CD68^+^ Mφs, CD15^+^ neutrophils, CD11b^+^ myeloid cells and CD8^+^ T cells in the nontumor (NT) and intratumor (IT) regions of hepatocellular carcinoma (HCC). **(A)** Representative immunohistochemistry images of CD68^+^ Mφs, CD15^+^ neutrophils, CD11b^+^ myeloid cells and CD8^+^ T cells in human HCC. **(B)** The numbers of CD68^+^ Mφs, CD15^+^ neutrophils, CD11b^+^ myeloid cells and CD8^+^ T cells in the NT (upper) and IT regions (bottom) of human HCC. Cell numbers were calculated as the cell count per ×400 field. Data are expressed as the mean ± SEM. Student’s *t* tests were applied, and ****P < 0.0001. ns, not significant.

To investigate the correlation between serum CRP and *in situ* immune infiltration in HCC, we performed immuno-histochemistry to assess the distribution of CD11b^+^ myeloid cells, CD68^+^ TAMs, CD15^+^ TANs and CD8^+^ T cells in the nontumor (NT) and intratumor (IT) regions of HCC tissues. Clear and distinguishable staining was observed for all of the immune cell markers (**Figure 2A**). Statistics showed that the numbers of TAMs and TANs in the NT region, but not IT region, of HCC tissues were significantly higher in patients with an elevated serum CRP level than in those with a low CRP level (both *p* < 0.0001; **Figure 2B**), whereas no difference in the infiltration of CD8^+^ T cells or CD11b^+^ cells was observed in either NT or IT regions (**Figure 2B**). Subgroup analyses of AST concentration further supported the association of serum CRP levels with TAM and TAN densities in the NT region of HCC tissues (**Table 2**). Taken together, these results indicate that elevated serum CRP level correlated with increased TAM and TAN infiltration in the NT region of HCC independent of liver function.

**Table 2:** The relationship between serum CRP and immune cell densities of HCC patients.

		Low AST			High AST			Total		
		CRP			CRP			CRP		
		Low	High	*p*	Low	High	*p*	Low	High	*p*
CD68*_N_*	Low	69	31	**0.005**	38	38	**0.04**	107	72	**<0.001**
	High	34	37		27	53		60	90
CD68*_T_*	Low	42	19	**0.04**	18	20	0.412	60	39	0.029
	High	58	52		47	71		107	123
CD15*_N_*	Low	59	39	**0.002**	39	37	**0.014**	97	58	**<0.001**
	High	39	44		24	52		64	95
CD15*_T_*	Low	55	30	0.128	29	36	0.053	84	65	0.096
	High	43	38		34	52		77	87

AST, Aspartate aminotransferase; N, nontumor; T, intratumor.

Chi-square test or Fisher’s exact test was used, and p < 0.05 was considered statistically significant and is shown in bold.

### Circulating CRP Level and Mφ Subtypes in HCC

**Figure 3:**
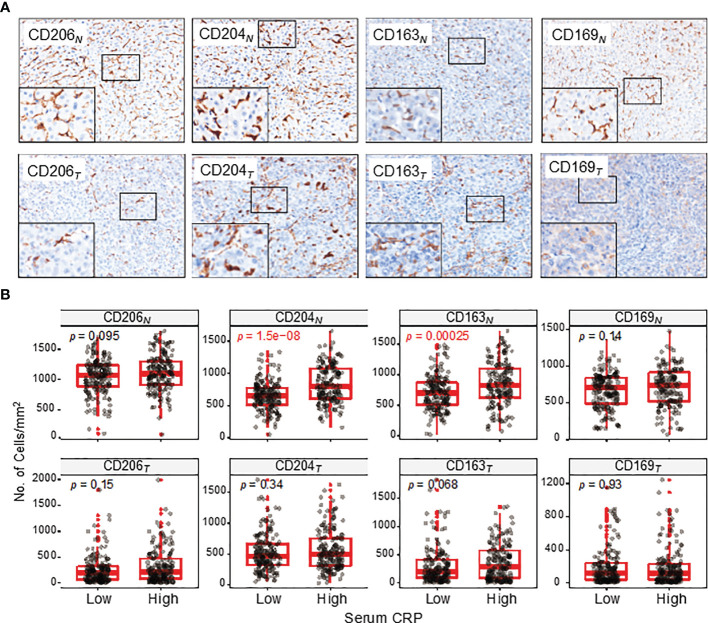
CD206^+^, CD204^+^, CD163^+^ and CD169^+^ Mφ distributions in the NT and IT regions of HCC. **(A)** Representative immunohistochemistry images of CD206^+^, CD204^+^, CD163^+^ and CD169^+^ Mφs in the NT (upper) and IT (bottom) regions of HCC. **(B)** The numbers of CD206^+^, CD204^+^, CD163^+^ and CD169^+^ Mφs in the NT (upper) and IT (bottom) regions of HCC. Cell numbers were calculated as the cell count per ×400 field. Data are expressed as the mean ± SEM. Student’s *t* tests were applied.

Mφs are versatile plastic cells that have multiple functions and phenotypes in response to environmental signals (15–17). To assess different subtypes of Mφ and their correlations with serum CRP level, we examined 4 common markers that are mainly expressed on Mφs (**Figure 3A**). Of these, CD206^+^, CD204^+^ and CD163^+^ Mφs are suggested to represent immunosuppressive Mφs that promote tumor progression, whereas CD169^+^ Mφs display antitumor properties in HCC (18–20). The results showed that the densities of CD204^+^ and CD163^+^ Mφs in the NT region of HCC tissues were significantly higher in patients with an elevated serum CRP level than in those with a lower level (*p* < 0.0001 and *p* = 0.0003 for CD204^+^ and CD163^+^ Mφs, respectively; **Figure 3B**). No significant correlation was found between CD206^+^ or CD169^+^ Mφ densities and serum CRP levels in either NT or IT regions. Collectively, these results suggest that serum CRP levels might be an indicator of an increased density of immunosuppressive Mφs in HCC tissues.

### CRP Expression and Immune Status in HCC

**Figure 4:**
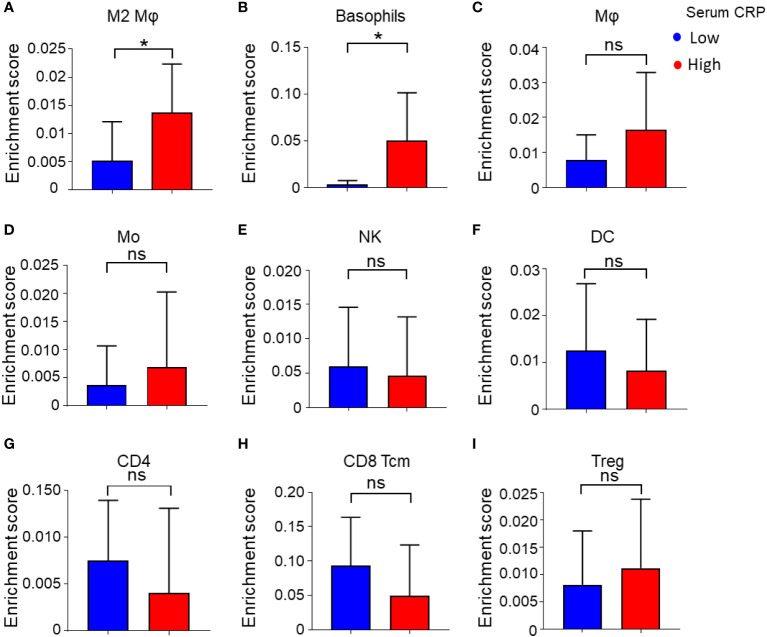
Enrichment score of 34 immune cell subtypes calculated by xCell. **(A–I)** Immune cell enrichment score of M2 Mφs **(A)**, basophils **(B)**, Mφs **(C)**, monocytes (Mo) **(D)**, NK cells **(E)**, DCs **(F)**, CD4^+^ T cells **(G)**, CD8^+^ central memory T cells (Tcm) **(H)**, Tregs **(I)**, and between low and high serum CRP level groups. Student’s *t* tests were applied, and *P < 0.05. ns, not significant.

To further characterize the association of serum CRP level and the immune status of the tumor microenvironment (TME) in HCC tissues, we analyzed gene expression microarray data of 24 HCC samples from our previous study (21). Serum CRP data were available for 17 samples, and the samples were divided into two groups with the same cutoff value (**Figure 1A**, red dots). The overall type-specific enrichment scores for 34 types of immune cells based on transcriptome data were calculated by xCell, a gene signature-based method. Statistics showed that tumor tissues from patients with higher levels of serum CRP had higher infiltration of myeloid cells, such as alternatively activated Mφs (M2, *p* = 0.04; **Figure 4A**) and basophils (*p* = 0.02; **Figure 4B**), while no significant difference in the enrichment score of other major immune cell types was found (**Figures 4C–I** and **Supplementary Table 2**).

**Figure 5:**
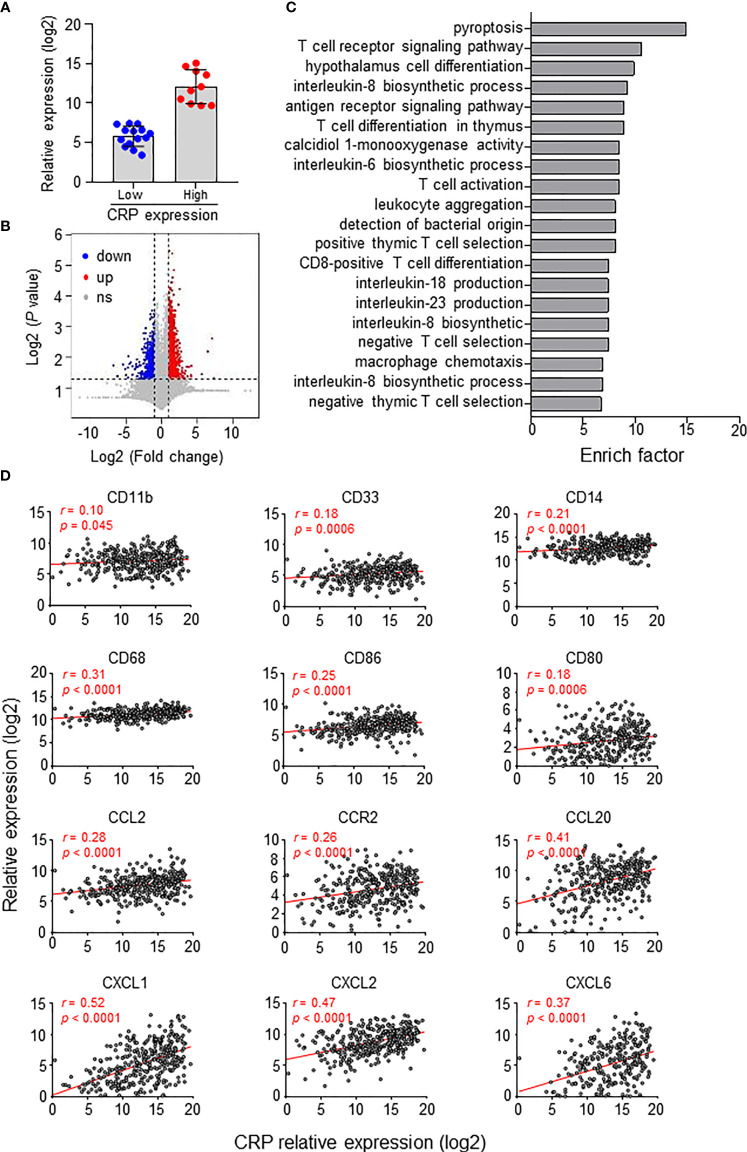
CRP expression *in situ* and immune status of HCC patients. **(A)** Twenty-four patients were divided into two groups based on *in situ* CRP expression, with 10 high-expressing and 14 low-expressing patients. **(B)** Volcano plot of gene expression. Upregulated genes (fold change > 2, *P* < 0.01, highlighted in red) and downregulated genes (fold change < 0.5, *P* < 0.01, highlighted in blue) were identified in the high CRP group compared to the low CRP group. **(C)** Top twenty GO terms from the GO enrichment analysis using the differentially expressed genes between the low CRP versus high CRP groups. **(D)** TCGA data showing the associations between the expression levels of CRP and myeloid-related genes in HCC tissues. Correlations were determined by Pearson correlation and linear regression analysis.

We also analyzed gene expression differences between groups divided by the CRP expression level in 24 HCC tumor tissues (**Figure 5A**). Overall, the CRP high-expressing group had 967 upregulated and 779 downregulated genes compared to the CRP low-expressing group (**Figure 5B**). Gene Ontology (GO) analysis of the differentially expressed genes showed that these genes were enriched in immune regulation processes, such as T cell signaling, leukocyte aggregation and Mφ chemotaxis (**Figure 5C**). Furthermore, analysis of 366 HCC tumor samples from TCGA demonstrated positive associations of tissue CRP level and genes related to myeloid cells, including CD68 and CD14, as well as chemokines and receptors for the chemotaxis of monocytes or neutrophils, such as CCL2, CCR2, and CXCL1 (**Figure 5D**). Collectively, these results suggest that CRP level positively correlate with myeloid cell infiltration in HCC tissues.

### Combined Prognostic Power of CRP Levels and TAM/TAN Density

**Figure 6:**
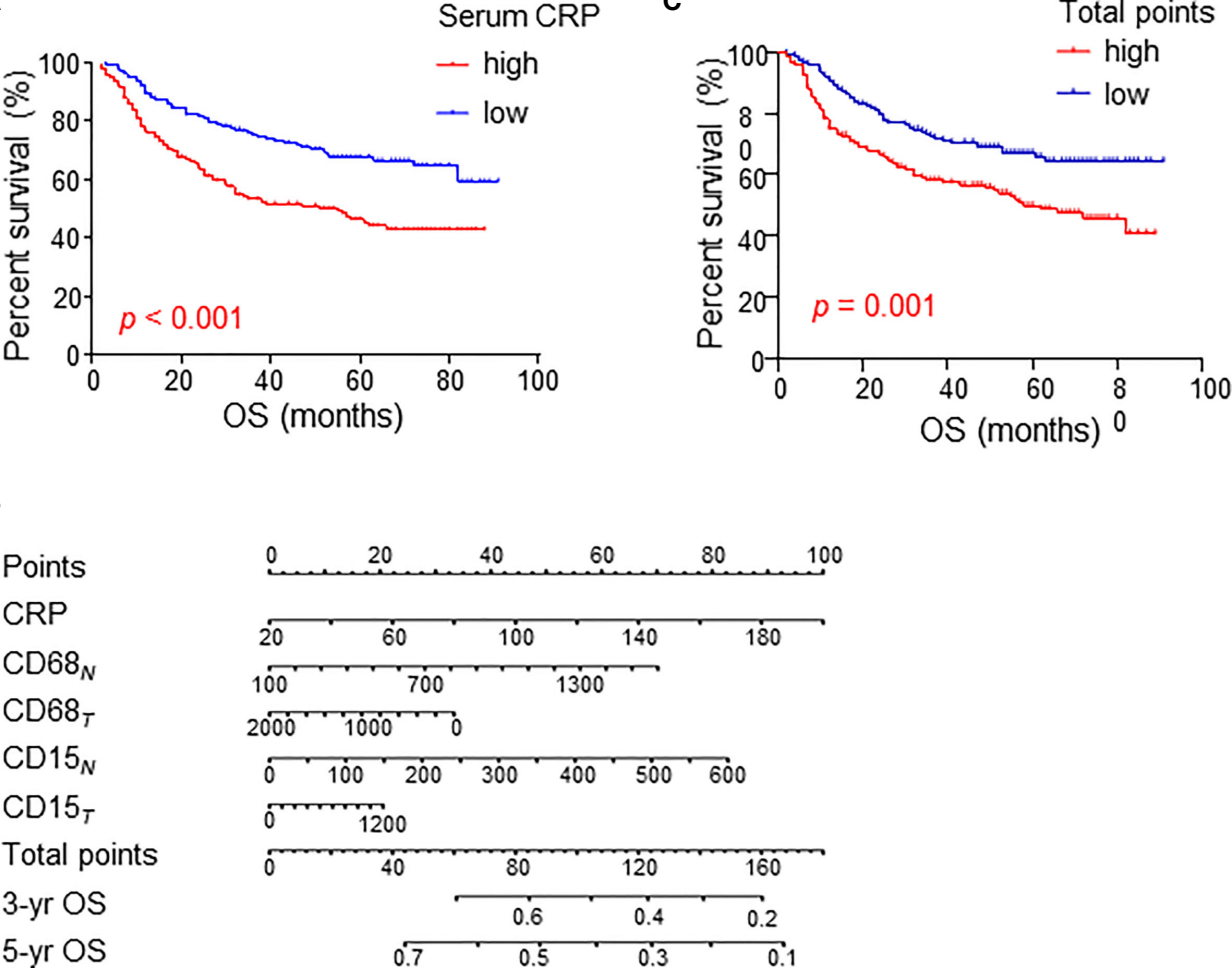
Cumulative overall survival curves of HCC patients grouped based on CRP levels and TAM/TAN densities in the NT or IT region. **(A)** Overall survival (OS) of HCC patients divided by serum CRP levels. **(B)** A nomogram to predict prognosis of HCC patients. **(C)** OS of HCC patients divided by total points of nomogram. OS was estimated using the Kaplan–Meier method and compared using the log-rank test. .

The above results suggest a relationship between serum CRP level and myeloid cell infiltration in HCC tissues. Next, we analyzed their clinical value in predicting patient prognoses in the cohort of 329 HCC patients. Consistent with previous reports from other groups, serum CRP levels alone were correlated with poor OS in HCC patients (**Figure 6A**).

A fitting model was presented with a nomogram to predict the prognosis of HCC patients using serum CRP level, tumor-infiltrating TAMs and TANs (**Figure 6B**). The total points of nomogram for each patient were calculated, and patients were divided into two groups according to the median value of total points. The Kaplan-Meier curves showed that the total points of nomogram could notably predict OS of HCC patients (**Figure 6C**). Multivariate analysis revealed that the total points of nomogram was an independent prognostic factor for OS (**Table 3**). In addition, the associations between the total points of nomogram and clinicopathological variables were analyzed, and the results showed that the total points of nomogram was significantly correlated with AST level, tumor number, tumor size and TNM stage (**Supplementary Tables 3**). Taken together, these results indicate that the combination of serum CRP levels with myeloid infiltrations is closely associated with disease progression and could represent a powerful criterion for predicting prognoses in HCC patients.

**Table 3:** Univariate and multivariate analyses of factors associated with patients’ overall survival.

Variables	Univariate			Multivariate		
	HR	95% CI	*P* value	HR	95% CI	*P* value
Age (> 50 vs. <= 50, years)	0.877	0.613-1.254	0.472			
TNM (II+III vs. I)	3.544	2.522-4.981	**<0.0001**	4.447	2.648-7.469	**<0.0001**
AST (> 40 vs. <= 40, U/L)	2.199	1.557-3.105	**<0.0001**	2.051	1.397-3.012	**<0.0001**
AFP (> 25 vs. <= 25, ng/mL)	2.506	1.691-3.715	**<0.0001**	2.536	1.655-3.887	**<0.0001**
ALT (> 37 vs. <= 37, U/L)	1.361	0.971-1.909	0.074			
TBIL (> 17.1 vs. <= 17.1, μmol/L)	0.849	0.573-1.257	0.414			
Tumor number (multiple vs. single)	2.555	1.802-3.622	**<0.0001**	0.529	0.308-0.907	**0.021**
Tumor size (> 4 vs. <= 4, cm)	1.618	1.226-2.135	**0.001**			
Total points (high vs. low)	1.777	1.242-2.543	**0.002**	1.519	1.046-2.205	**0.028**

AFP, alpha-fetoprotein; AST, aspartate aminotransferase; ALT, alanine aminotransferase; TBIL, total bilirubin.

Cox proportional hazards regression model; variables that were associated with overall survival in the univariate analysis were adopted as covariates in the multivariate analysis and were entered into the equation using the forward likelihood ratio method. P < 0.05 was considered statistically significant and is shown in bold.

## Discussion

Serum CRP has been widely used as a highly sensitive biomarker of systemic inflammation in clinical settings. In the present study, we applied *in situ* immunostaining and gene expression analysis and found that patients with elevated serum CRP levels often have high densities of CD68^+^ TAMs and CD15^+^ TANs in HCC tissues. These myeloid cells displayed immunosuppressive properties, and the combination of serum CRP with TAMs or TANs could represent a powerful criterion for predicting prognoses in HCC patients. These findings reveal a novel biological value of serum CRP and suggest that the evaluated level of serum CRP might serve as a potential indicator of immunosuppressive myeloid cell infiltration in HCC tissue.

CRP is an acute phase inflammatory marker of systemic inflammation that is mainly produced by hepatocytes in response to infection, liver injury or inflammatory cytokine release. Over the past few decades, emerging evidence has shown that serum CRP is associated with the risk of several chronic conditions, such as cardiovascular disease, atherosclerosis and cancer (3, 22, 23). The clinical value of serum CRP has also been explored in tumor settings, such as ovarian cancer, breast cancer, colorectal cancer, lung cancer, prostate cancer, renal cell carcinoma, and pancreatic cancer (24–31). In patients with HCC, several studies have reported that high serum CRP levels predict poor clinical outcomes in the case of both resectable and unresectable tumors (4, 5, 32, 33). Although the predictive power of CRP has been intensely investigated, its biological significance and underlying mechanisms are not fully understood. In the present study, we confirmed the negative prognostic role of CRP in HCC. Furthermore, we found that serum CRP was closely associated with the immune status of the TME. Specifically, patients with higher serum CRP levels often had more TAMs and TANs infiltrating the TME. Due to the tumor-promoting roles of TAMs and TANs, these results might partially explain why serum CRP is an indicator of a progressive tumor.

Tumor-associated myeloid cells, including TAMs and TANs, are major immunosuppressive components of the TME and contribute to tumor progression by promoting genetic instability, nurturing cancer stem cells, supporting metastasis, and suppressing protective adaptive immunity (34). We and other groups have demonstrated that the number of CD68^+^ TAMs in HCC is negatively correlated with patient prognosis (11, 35), and TAN density is also associated with poor prognosis (36–38). The present study revealed the tight association of serum CRP with TAMs and TANs infiltrating the tissues, especially TAMs expressing CD204 and CD163 with immunosuppressive properties. This conclusion is supported by the following observations: 1) patients with elevated serum CRP levels were likely to have more CD204^+^ and CD163^+^ tissue-infiltrating Mφs; 2) serum CRP selectively correlated with enrichment scores of M2 Mφs and basophils from the gene expression microarray; and 3) CRP expression also positively correlated with chemokines and chemokine receptors associated with myeloid cell mobilization. It has been reported that CRP was mainly expressed by hepatocytes in response to inflammatory factors, and could promote the formation of immunosuppressive immune cells (6, 39, 40). Together with our findings, it is possible that the secreted inflammatory cytokines in HCC tissues could upregulate the synthesis and secretion of CRP by hepatocytes, which further promote the accumulation of myeloid cells. The regulating mechanisms of CRP secretion and myeloid cell infiltration deserve further investigations.

We noted that serum CRP correlated with TAMs and TANs in the NT but not the IT regions of HCC tissues, suggesting that the tumor phenotype is not only dictated by genetic and epigenetic alterations in the tumor cells *per se* but is also influenced by molecular crosstalk between tumor cells and the surrounding microenvironment, especially the infiltrating immune cells. This is particularly relevant for liver cancer, where the majority of HCCs (up to 90%) develop in the background of chronic liver inflammation caused by hepatitis B or C virus, alcoholic steatohepatitis, or nonalcoholic steatohepatitis (41). Accordingly, it has been reported that a predominant Th2-like cytokine profile occurs in the liver milieu and that a shift toward anti-inflammatory/immunosuppressive responses plays a vital role in promoting tumor progression and venous metastases of HCC (42). Our previous studies have also revealed that monocytes/Mφs in the peritumoral region of HCC could effectively suppress tumor-specific T cell immunity *via* PD-L1-PD1 and induce cancer cell autophagy in the invading edge region by secreting TNF-α and IL-1β, which resulted in epithelial-mesenchymal transition of cancer cells and tumor metastasis (11, 13). These results suggest the importance of the liver microenvironment in the aggressive malignancy of HCC. However, the context of immune reactions in the TME is tissue specific and depends on multiple factors. How to manipulate local inflammation, thus lead to the reactivation of immune status in HCC tissue deserves further study.

In conclusion, our results provide insights into the association between serum CRP and myeloid cell infiltration in HCC. Given the increasing appreciation of the TME as a critical regulator of tumor immunity and immunotherapy (43, 44), the serum CRP level might serve as a simple indicator of the immune status in the tumor site and thus has a potential application for treatment strategies targeting the immunosuppressive TME for patients with HCC.

## Data Availability Statement

The raw data supporting the conclusions of this article will be made available by the authors, without undue reservation.

## Ethics Statement

The studies involving human participants were reviewed and approved by Institutional Review Board of Sun Yat-sen University Cancer Center. The patients/participants provided their written informed consent to participate in this study. Written informed consent was obtained from the individual(s) for the publication of any potentially identifiable images or data included in this article.

## Author Contributions

YW designed research. YW, ZL, and ZH performed research. YW analyzed data. XY and ZH collected human samples. YW, LZ, and JX wrote the paper. All authors contributed to the article and approved the submitted version.

## Funding

This work was supported by project grants from the National Key R&D Program of China (2017YFA0505803, 2021YFC2300601), and the National Natural Science Foundation of China (81772536).

## Conflict of Interest

The authors declare that the research was conducted in the absence of any commercial or financial relationships that could be construed as a potential conflict of interest.

## Publisher’s Note

All claims expressed in this article are solely those of the authors and do not necessarily represent those of their affiliated organizations, or those of the publisher, the editors and the reviewers. Any product that may be evaluated in this article, or claim that may be made by its manufacturer, is not guaranteed or endorsed by the publisher.
