# Corrigendum: C-reactive protein is an indicator of the immunosuppressive microenvironment fostered by myeloid cells in hepatocellular carcinoma

**DOI:** 10.3389/fonc.2023.1280727

**Published:** 2023-10-24

**Authors:** Yongchun Wang, Zhixiong Li, Zhijie Huang, Xingjuan Yu, Limin Zheng, Jing Xu

**Affiliations:** ^1^ Collaborative Innovation Center for Cancer Medicine, State Key Laboratory of Oncology in South China, Sun Yat-sen University Cancer Center, Guangzhou, China; ^2^ Ministry Of Education (MOE) Key Laboratory of Gene Function and Regulation, School of Life Science, Sun Yat-sen University, Guangzhou, China

**Keywords:** CRP, HCC, immune microenviroment, tumor associated macrophage, tumor associated neutrophil

## Error in Figure/Table

In the published article, there was an error in **Figure 4** as published. The figure showed that the abundance of immune cell subsets in tumor tissue of patients with high or low serum CRP levels. However, during the editing process of the figure, we inadvertently entered the wrong values of cell subset-Mo, NK, DC, CD4, CD8 Tcm and Treg, which resulted in the error of **Figures 4D–I**. The corrected **Figure 4** and its caption appear below.

**Figure 4:**
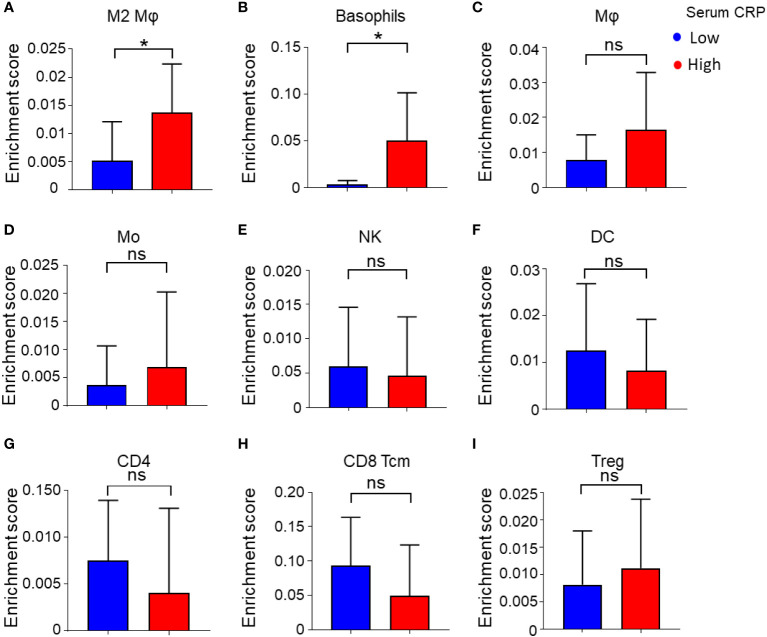
Enrichment score of 34 immune cell subtypes calculated by xCell. **(A–I)** Immune cell enrichment score of M2 Mφs **(A)**, basophils **(B)**, Mφs **(C)**, monocytes (Mo) **(D)**, NK cells **(E)**, DCs **(F)**, CD4+ T cells **(G)**, CD8+ central memory T cells (Tcm) **(H)**, Tregs **(I)**, and between low and high serum CRP level groups. Student’s t tests were applied, and *P < 0.05. ns, not significant.

The authors apologize for this error and state that this does not change the scientific conclusions of the article in any way. The original article has been updated.

