# Supplementary material for: C-Reactive Protein Is an Indicator of the Immunosuppressive Microenvironment Fostered by Myeloid Cells in Hepatocellular Carcinoma
Source: Front Oncol. 2022 Jan 6;11:774823. doi: 10.3389/fonc.2021.774823 (PMC8770831; doi:10.3389/fonc.2021.774823)
Supplement: Supplementary file 1 [file Table_1.docx]

| Supplementary Table 1. The relationship between serum CRP and patients’ clinicopathological features | | | | |
| --- | --- | --- | --- | --- |
| Characteristics | | Serum CRP | |  |
|  |  | Low (n, %) | High (n, %) | *p* |
| Age | < 55 | 103 (31.3) | 113 (34.3) | 0.216 |
|  | ≥ 55 | 62 (18.8) | 51 (15.5) |  |
| Gender | male | 147 (44.7) | 141 (42.9) | 0.329 |
|  | female | 18 (5.5) | 23 (7.0) |  |
| HBV infection | no | 34 (11.9) | 12 (4.2) | **0.001** |
|  | yes | 112 (39.3) | 127 (44.6) |  |
| BCV infection | no | 157 (49.2) | 159 (49.8) | 0.083 |
|  | yes | 3 (0.9) | 0 (0) |  |
| AFP (ng/mL) | ≤ 25 | 66 (20.7) | 56 (17.6) | 0.399 |
|  | > 25 | 97 (30.4) | 100 (31.3) |  |
| Child-Pugh | A | 157 (49.4) | 148 (46.5) | 0.476 |
|  | B | 5 (1.6) | 7 (2.2) |  |
|  | C | 0 (0) | 1 (0.3) |  |
| Tumor number | single | 137 (41.9) | 110 (33.6) | **< 0.001** |
|  | multiple | 26 (8.0) | 54 (16.5) |  |
| Tumor size (cm) | ≤ 5 | 69 (21.3) | 24 (7.4) | **< 0.001** |
|  | > 5 | 94 (29.0) | 137 (42.3) |  |
| TNM | I+II | 129 (40.6) | 84 (26.4) | **< 0.001** |
|  | III+IV | 33 (10.4) | 72 (22.6) |  |
| AST (U/L) | ≤ 40 | 98 (30.0) | 73 (22.3) | **0.005** |
|  | > 40 | 65 (19.9) | 91 (27.8) |  |
| ALT (U/L) | ≤ 40 | 99 (30.4) | 94 (28.8) | 0.573 |
|  | > 40 | 64 (19.6) | 69 (21.2) |  |
| TBIL (μmol/L) | ≤ 17.1 | 119 (36.4) | 119 (36.4) | 0.928 |
|  | > 17.1 | 44 (13.5) | 45 (13.8) |  |

Abbreviation: AST, aspartate aminotransferase; ALT, alanine aminotransferase; TBIL, total bilirubin; AFP, alpha-fetoprotein.

Note: Chi-square test or Fisher’s exact test was used, and *p* < 0.05 was considered statistically significant and showed in bold.

| Supplementary Table 2. Immune cell enrichment scores between serum CRP levels of patients with HCC | | | |
| --- | --- | --- | --- |
|  | Serum CRP High | Serum CRP Low | *p* value |
|  | Mean ± SD | Mean ± SD |  |
| ImmuneScore | 0.062 ± 0.083 | 0.110 ± 0.077 | 0.236 |
| CD4 T-cell subpopulations |  |  |  |
| CD4+ T-cells | 0.004 ± 0.009 | 0.008 ± 0.006 | 0.373 |
| CD4+ Tcm | 0.012 ± 0.016 | 0.005 ± 0.010 | 0.298 |
| CD4+ Tem | 0.001 ± 0.002 | 0.001 ± 0.002 | 0.945 |
| CD4+ memory T-cells | 0.006 ± 0.016 | 0.016 ± 0.012 | 0.172 |
| CD4+ naive T-cells | 0.006 ± 0.012 | 0.010 ± 0.019 | 0.693 |
| Th1 cells | 0.008 ± 0.009 | 0.016 ± 0.010 | 0.105 |
| Th2 cells | 0.023 ± 0.045 | 0.066 ± 0.068 | 0.148 |
| Tregs | 0.011 ± 0.013 | 0.008 ± 0.010 | 0.589 |
| CD8 T-cell subpopulations |  |  |  |
| CD8+ T-cells | 0.030 ± 0.040 | 0.053 ± 0.041 | 0.267 |
| CD8+ Tcm | 0.049 ± 0.074 | 0.094 ± 0.070 | 0.225 |
| CD8+ Tem | 0.017 ± 0.037 | 0.036 ± 0.030 | 0.252 |
| CD8+ naive T-cells | 0.001 ± 0.001 | 0.002 ± 0.003 | 0.296 |
| Tgd cells | 0.003 ± 0.007 | 0.004 ± 0.005 | 0.561 |
| NKT | 0.008 ± 0.008 | 0.010 ± 0.012 | 0.669 |
| NK cells | 0.005 ± 0.009 | 0.006 ± 0.009 | 0.738 |
| B-cell subpopulations |  |  |  |
| B-cells | 0.024 ± 0.045 | 0.067 ± 0.074 | 0.180 |
| Class-switched memory B-cells | 0.010 ± 0.011 | 0.021 ± 0.021 | 0.240 |
| Memory B-cells | 0.013 ± 0.024 | 0.028 ± 0.029 | 0.263 |
| pro B-cells | 0.001 ± 0.003 | 0.005 ± 0.007 | 0.180 |
| Plasma cells | 0.004 ± 0.003 | 0.015 ± 0.012 | **0.027** |
| naive B-cells | 0.005 ± 0.010 | 0.010 ± 0.020 | 0.530 |
| Monocyte/ Mφ subpopulations |  |  |  |
| Monocytes | 0.007 ± 0.013 | 0.004 ± 0.007 | 0.534 |
| Macrophages | 0.016 ± 0.017 | 0.008 ± 0.007 | 0.182 |
| Macrophages M1 | 0.013 ± 0.019 | 0.009 ± 0.007 | 0.492 |
| Macrophages M2 | 0.014 ± 0.009 | 0.005 ± 0.007 | **0.040** |
| DC subpopulations |  |  |  |
| DC | 0.008 ± 0.011 | 0.013 ± 0.014 | 0.505 |
| Activated DC | 0.078 ± 0.082 | 0.127 ± 0.072 | 0.204 |
| Plasmacytoid DC | 0.017 ± 0.031 | 0.020 ± 0.019 | 0.842 |
| Conventional DCs | 0.041 ± 0.034 | 0.034 ± 0.041 | 0.694 |
| Immature DC | 0.060 ± 0.060 | 0.087 ± 0.102 | 0.526 |
| Granulocyte subpopulations |  |  |  |
| Neutrophils | 0.000 ± 0.000 | 0.000 ± 0.000 | 0.304 |
| Basophils | 0.050 ± 0.052 | 0.003 ± 0.005 | **0.016** |
| Eosinophils | 0.001 ± 0.003 | 0.000 ± 0.000 | 0.304 |
| Mast cells | 0.002 ± 0.004 | 0.002 ± 0.003 | 0.836 |

Note: Students’*t* test was used, and *p* < 0.05 was considered statistically significant and showed in bold.

| Supplementary Table 3. The relationship between the total points of nomogram and patients’ clinicopathological features | | | | |
| --- | --- | --- | --- | --- |
| Characteristics | | Total points | |  |
|  |  | Low (n, %) | High (n, %) | *p* |
| Age (years) | < 55 | 95 (30.8) | 103 (33.4) | 0.341 |
|  | ≥ 55 | 59 (19.2) | 51 (16.6) |  |
| Gender | male | 131 (42.5) | 139 (45.1) | 0.166 |
|  | female | 23 (7.5) | 15 (4.9) |  |
| HBV infection | no | 22 (8.2) | 19(7.1) | 0.843 |
|  | yes | 118 (44.0) | 109 (40.7) |  |
| HCV infection | no | 147 (49.3) | 148 (49.7) | 0.084 |
|  | yes | 3 (1.0) | 0 (0) |  |
| AFP (ng/mL) | ≤ 25 | 63 (20.9) | 53 (17.5) | 0.275 |
|  | > 25 | 89 (29.5) | 97 (32.1) |  |
| Child-Pugh | A | 145 (48.2) | 143 (47.5) | 0.511 |
|  | B | 5 (1.7) | 7 (2.3) |  |
|  | C | 1 (0.3) | 0 (0) |  |
| Tumor number | single | 126 (41.2) | 110 (35.9) | **0.029** |
|  | multiple | 27 (8.8) | 43 (14.1) |  |
| Tumor size (cm) | ≤ 5 | 62 (20.5) | 28 (9.2) | **< 0.001** |
|  | > 5 | 89 (29.4) | 124 (40.9) |  |
| TNM | I+II | 114 (37.9) | 90 (29.9) | **0.004** |
|  | III+IV | 37 12.3) | 60 (19.9) |  |
| AST (U/L) | ≤ 40 | 91 (29.7) | 71 (23.2) | **0.022** |
|  | > 40 | 62 (20.3) | 82 (26.8) |  |
| ALT (U/L) | ≤ 40 | 91 (29.8) | 87 (28.5) | 0.594 |
|  | > 40 | 64 (19.6) | 69 (21.2) |  |
| TBIL (μmol/L) | ≤ 17.1 | 114 (37.3) | 108 (35.3) | 0.442 |
|  | > 17.1 | 39 (12.7) | 45 (14.7) |  |

Abbreviation: AST, aspartate aminotransferase; ALT, alanine aminotransferase; TBIL, total bilirubin; AFP, alpha-fetoprotein.

Note: Chi-square test or Fisher’s exact test was used, and *p* < 0.05 was considered statistically significant and showed in bold.
